# Supplementary material for: Single-chain dimers from de novo immunoglobulins as robust scaffolds for multiple binding loops
Source: Nat Commun. 2023 Sep 23;14:5939. doi: 10.1038/s41467-023-41717-5 (PMC10517939; doi:10.1038/s41467-023-41717-5)
Supplement: Supplementary file 1 — Supplementary information [file 41467_2023_41717_MOESM1_ESM.docx]

**
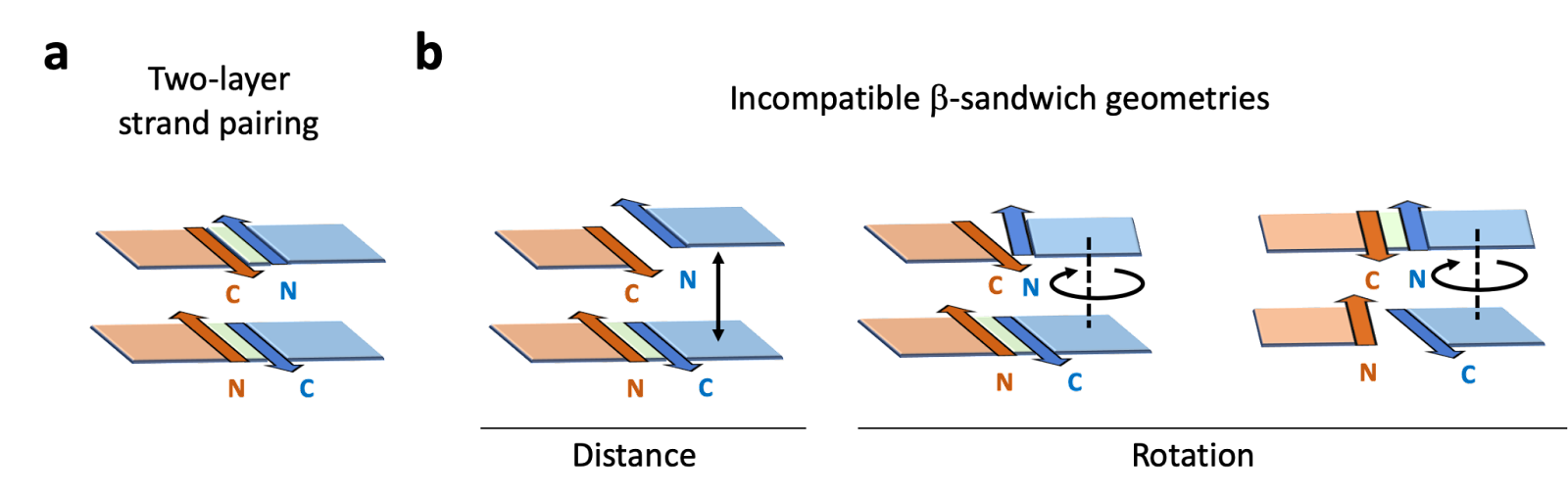
**

**Supplementary Figure 1. β-sandwich geometries compatible with two-layer strand pairing. a,** Optimal strand pairing in both layers is facilitated by Ig domains with similar β-sandwich geometries. **b**, Differences in the distance separation or rotations between the two opposing β-sheets in each domain hinder the formation of continuous hydrogen bond pairing in the two layers. Interdomain hydrogen-bonded pairing is colored in green.

**
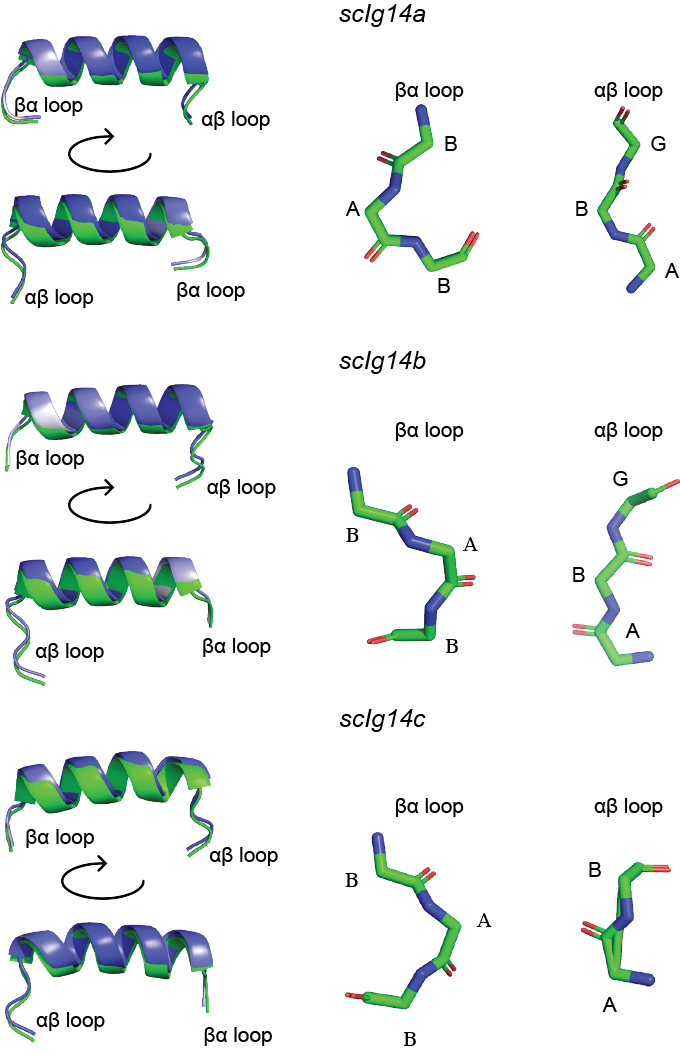
**

**Supplementary Figure 2. Connecting linkages between immunoglobulin domains of the selected 14-stranded parallel edge-to-edge designs.** On the left, first AlphaFold2^1^ model colored by increasing pLDDT (from red to blue; scale 70 to 100) superimposed to their corresponding designed helical linker. On the right, loop geometries described by ABEGO^2^ backbone torsion bins.


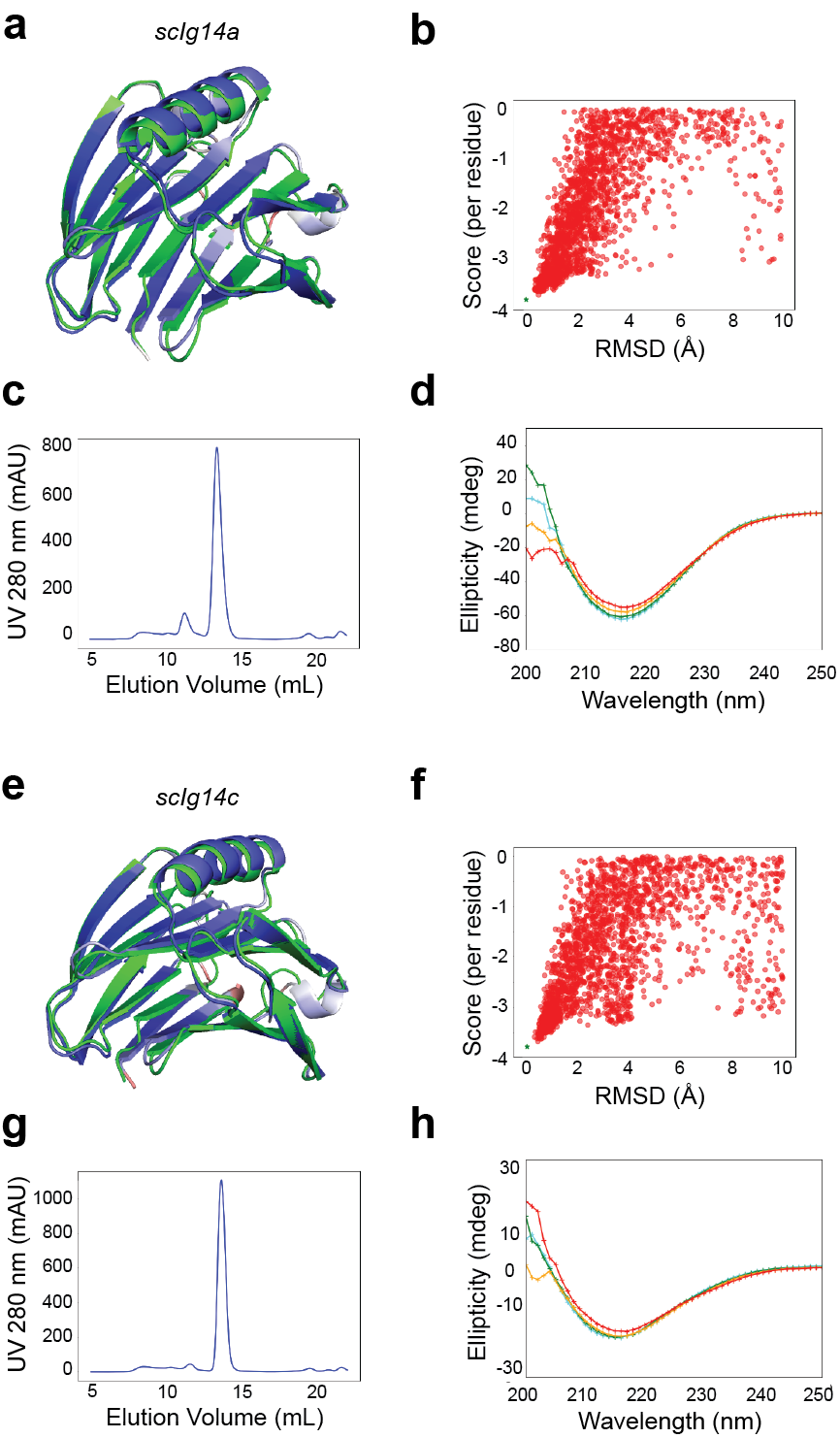


**Supplementary Figure 3. Design and experimental characterization of parallel, edge-to-edge single-chain immunoglobulin dimers.** **a,e.** First AlphaFold2^1^ model colored by increasing pLDDT (from red to blue; scale 70 to 100) superimposed to their corresponding design (scIg14a and scIg14c). **b,f.** Rosetta^3^ folding simulations^4^ for each of the designs. Y-axis indicates the per-residue Rosetta score and X-axis Cα-RMSDs against the design. Green stars correspond to the per-residue Rosetta score of the designs. Monodisperse **c,g.** Size-exclusion chromatograms of the expressed and purified single-chain dimers. **d,h.** Far-ultraviolet circular dichroism spectra at increasing temperatures (aqua: 25ºC, green: 40ºC, orange: 60ºC and red: 90ºC). Source data are provided as a Source Data file.


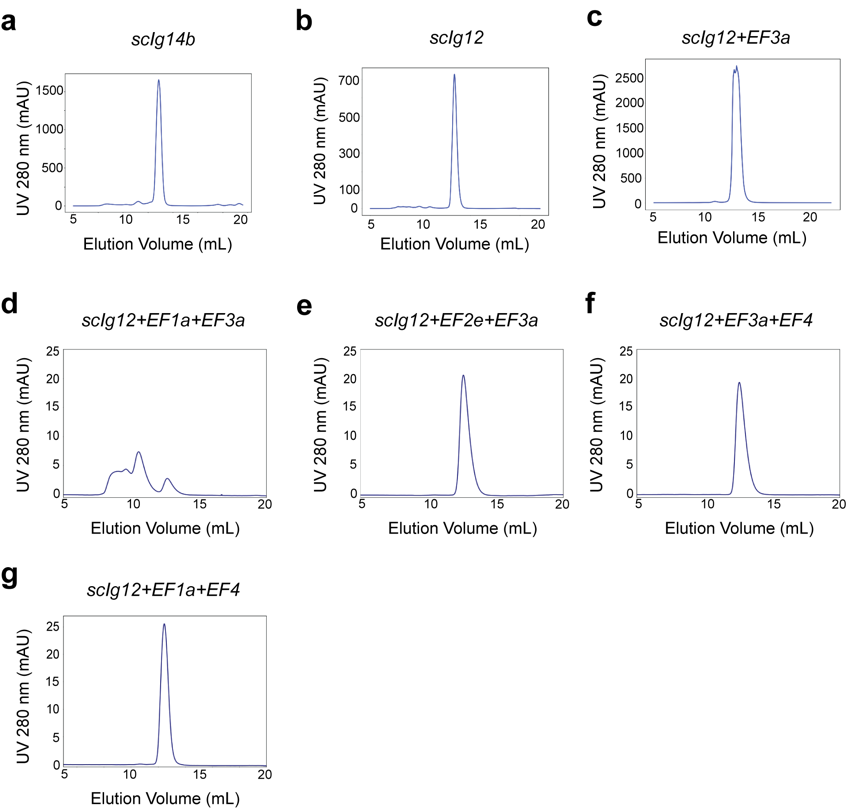


**Supplementary Figure 4. Size-exclusion chromatography. a,** scIg14b. **b,** scIg12. **c,** Monofunctional design of scIg12 (EF3a). **d-g,** bifunctional designs of scIg12: EF1a+EF3a (d), EF2e+EF3a (e), EF3a+EF4 (f) and EF1a+EF4 (g). Samples were run on a Superdex 75 10/300 increase and were prepared in 30 mM Tris·HCl, 250 mM sodium chloride, pH 8 at 25 ºC. All these chromatograms were obtained after affinity purification. Source data are provided as a Source Data file.


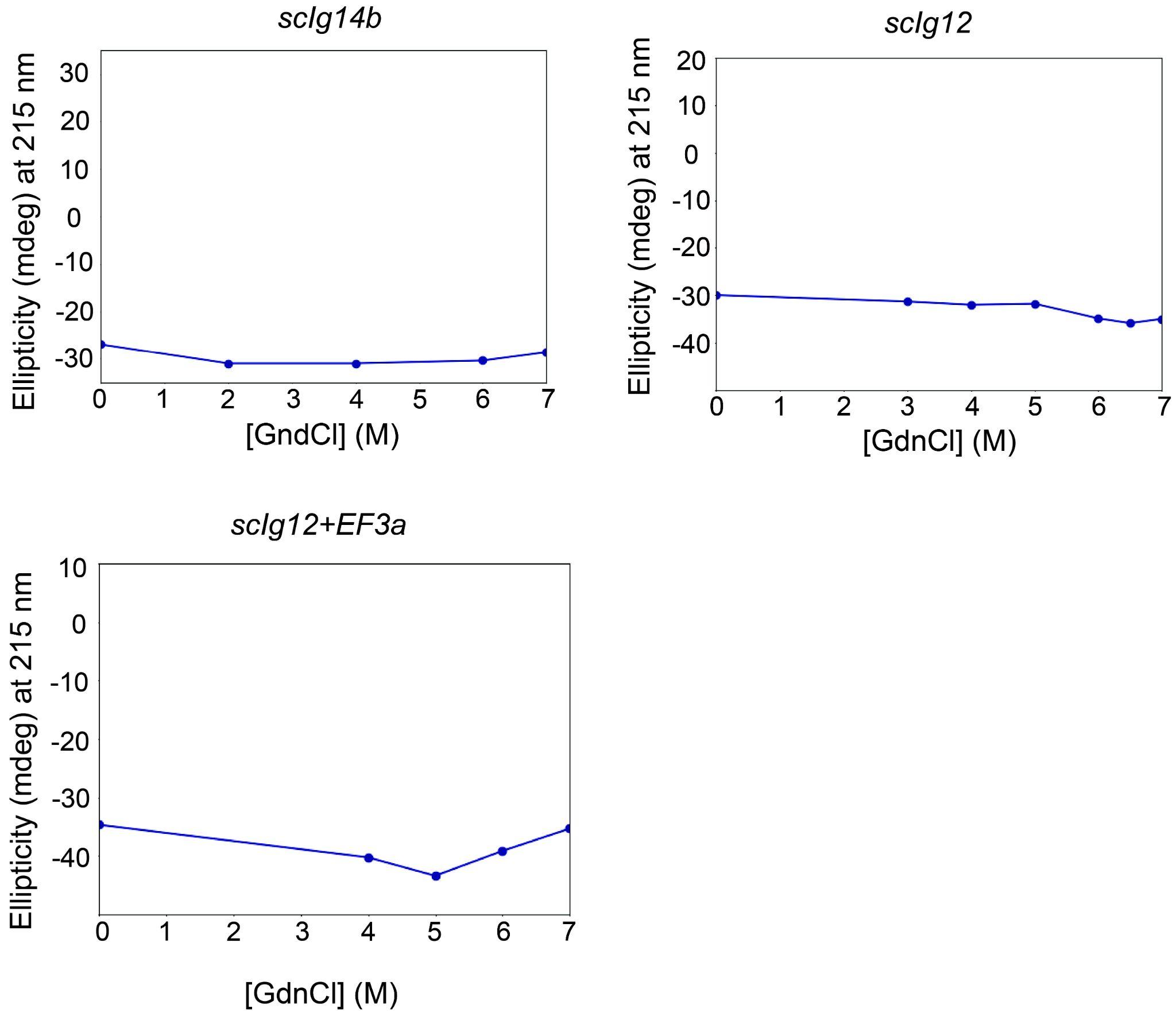


**Supplementary Figure 5. Chemical denaturation with GdnCl monitored with circular dichroism at 215 nm and 25 °C.** Source data are provided as a Source Data file.


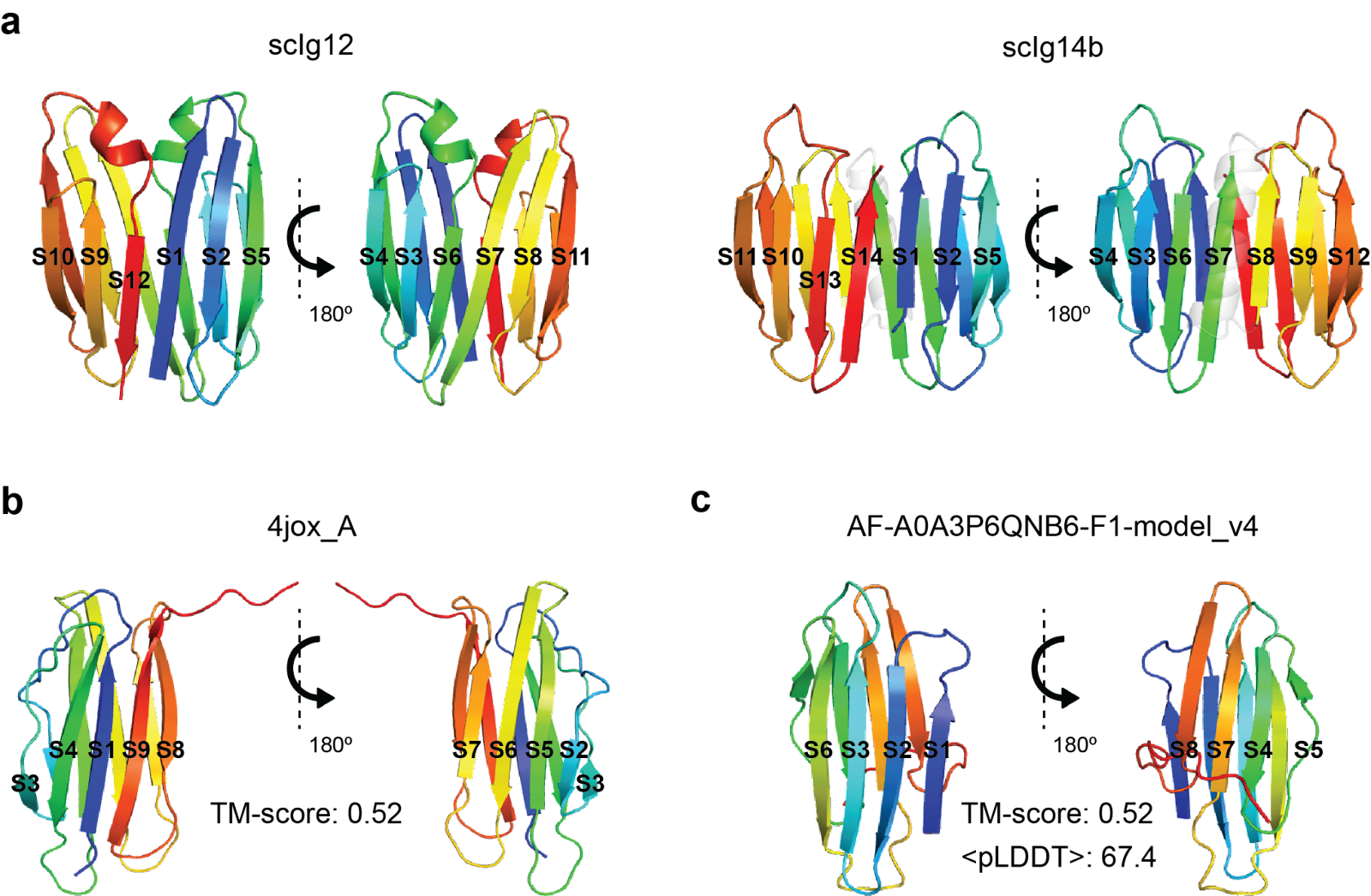


**Supplementary Figure 6. Top-ranked structural analogs of the 12- and 14-stranded single-chain Ig dimers in nature. a**, design models of scIg12 and a representative of the 14-stranded designs. **b,c**. Top hits either in the PDB (b) or the AlphaFold protein structure database^5^ (c), as retrieved from the FoldSeek server^6^. Closest analogs either to the 12-stranded or the 14-stranded are considered. All proteins are colored from the N- (blue) to C-termini (red), and β-strands numbered as S[1-14], where the number indicates the position in the sequence. TM-scores calculated with the TMalign software^7^ are provided. For the AlphaFold2^1^ model (c) the average pLDDT value is reported.


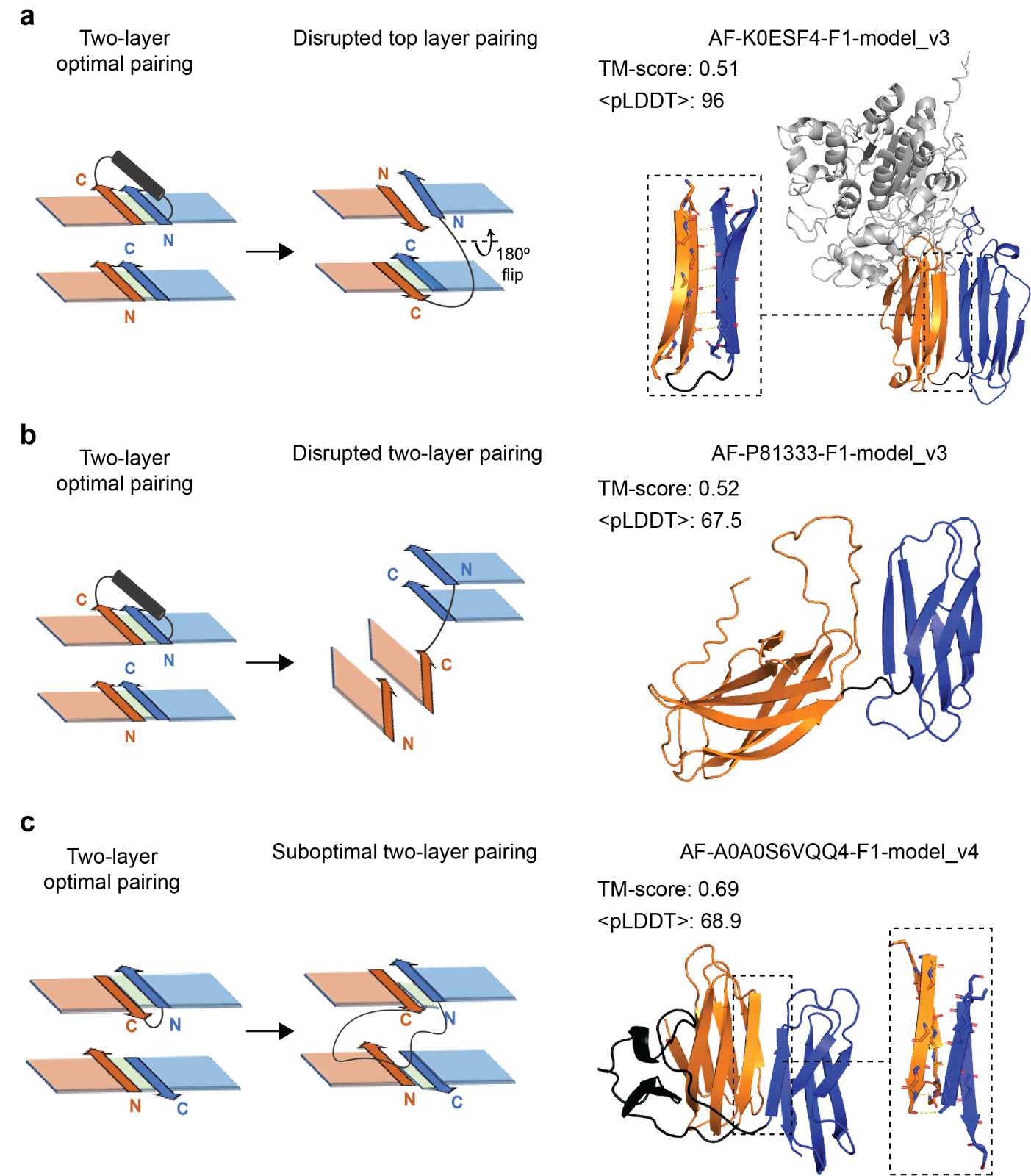


**Supplementary Figure 7. Structural analogs of the 12- and 14-stranded single-chain Ig dimers in nature with partially similar topologies.** Most similar structures in the AlphaFold protein structure database to either the 12-stranded or the 14-stranded topologies, as retrieved from the FoldSeek server^6^. From left to right, sketch highlighting topology changes with respect to the designs, and AlphaFold2 predicted models. **a**, single-chain dimer of 7-stranded Ig domains with an alternative interdomain connection (a β-arch cross-over) and disrupted strand pairing in one of the two layers. The rest of this multidomain in protein is colored in gray. **b**, two 7-stranded Ig domains fused through an extended linker and with an orientation incompatible with two-layer edge-to-edge pairing. **c**, domain structurally equivalent to scIg12 but with insertions in the N-termini and the interdomain connection (a subdomain colored in black). TM-scores (normalized with respect to the design length) and average pLDDT values are provided.

**
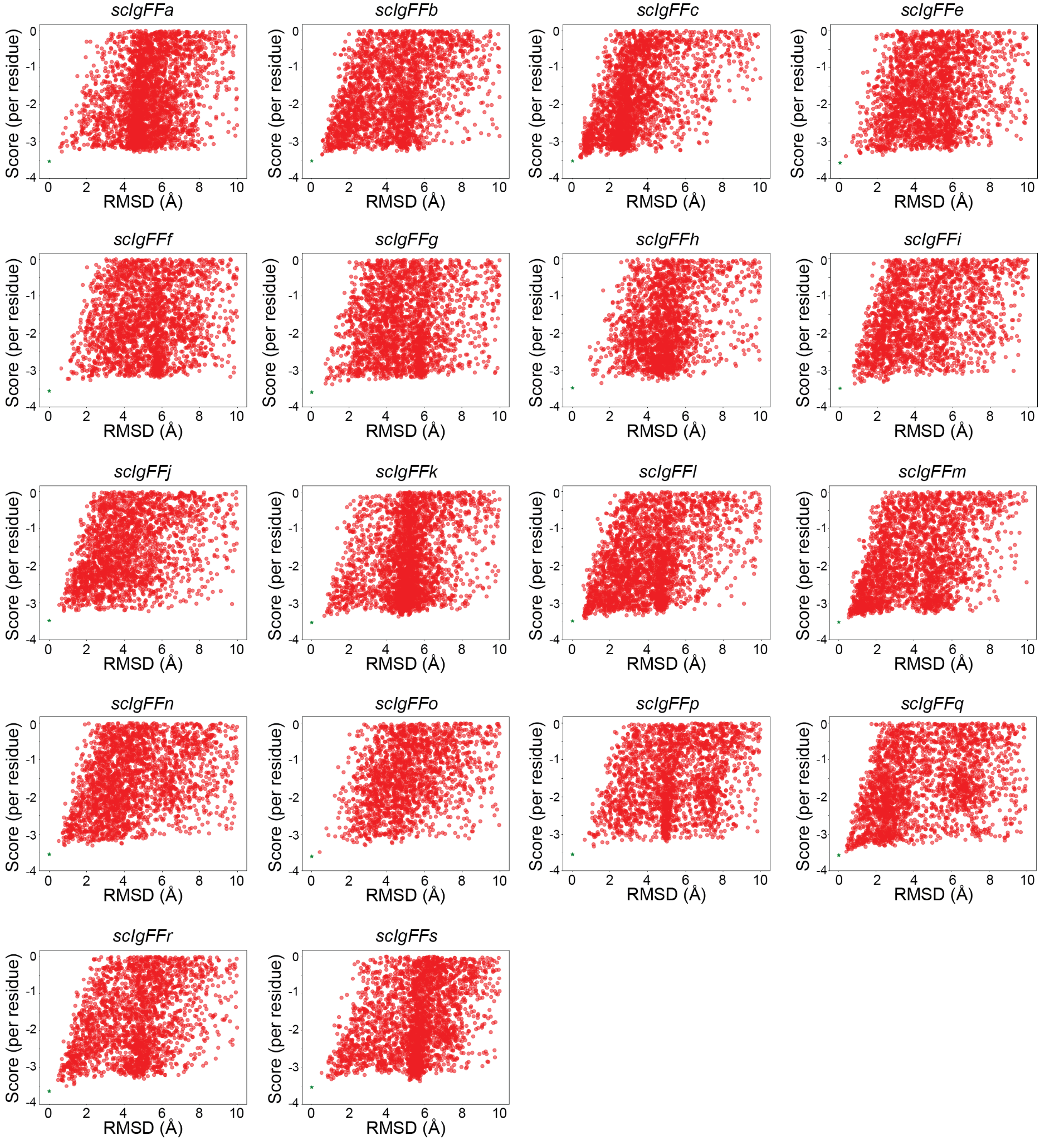
**

**Supplementary Figure 8. Folding simulations of 16 selected face-to-face single-chain immunoglobulin dimers.** Y-axis indicates the per-residue Rosetta score. The X-axis represents the RMSD against the design. Green stars correspond to the per-residue Rosetta score of the designs. Source data are provided as a Source Data file.


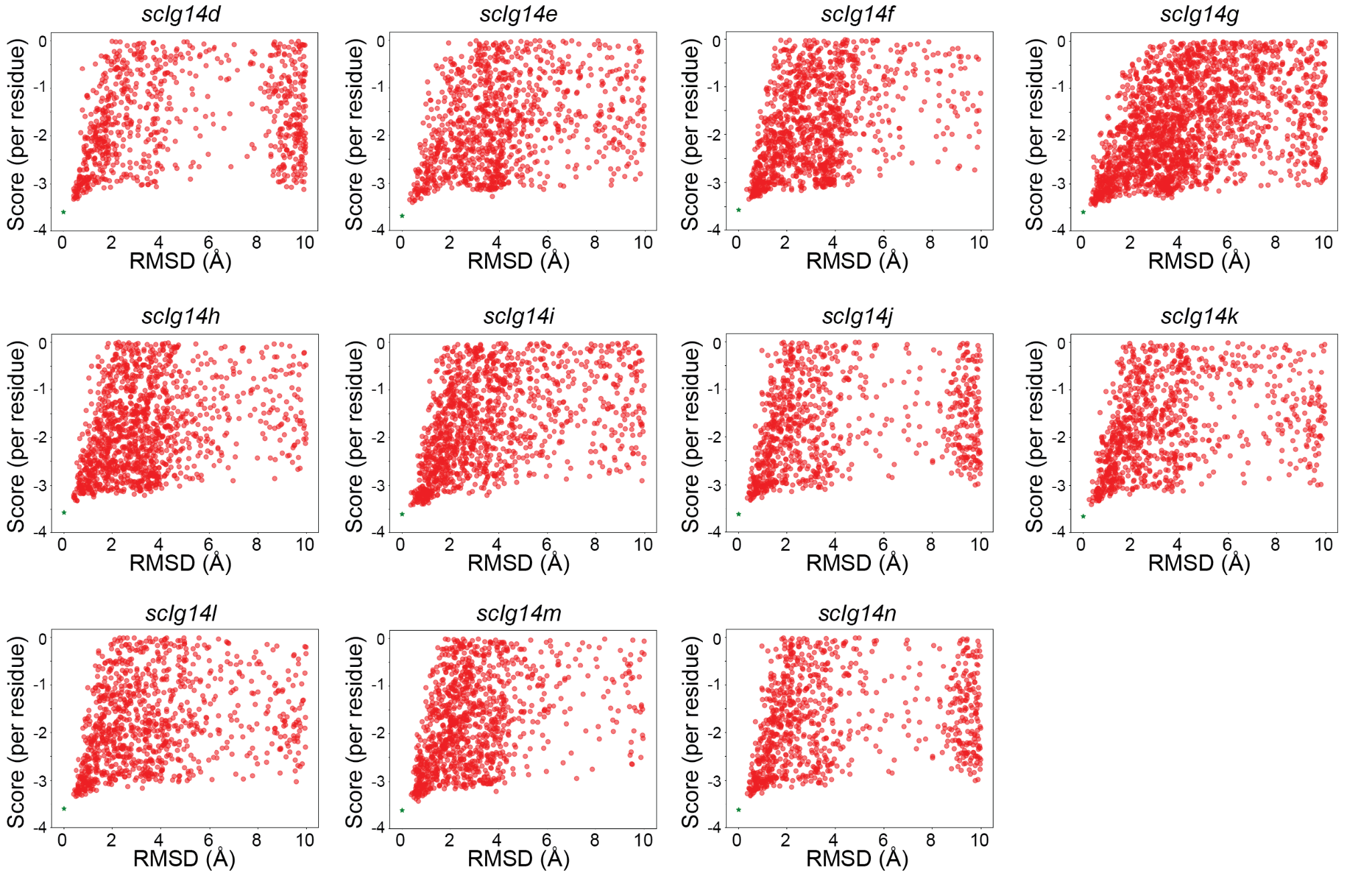


**Supplementary Figure 9. Folding simulations of 11 selected edge-to-edge parallel single-chain immunoglobulin dimers.** Y-axis indicates the per-residue Rosetta score. The X-axis represents the RMSD against the design. Green stars correspond to the per-residue Rosetta score of the designs. Source data are provided as a Source Data file.


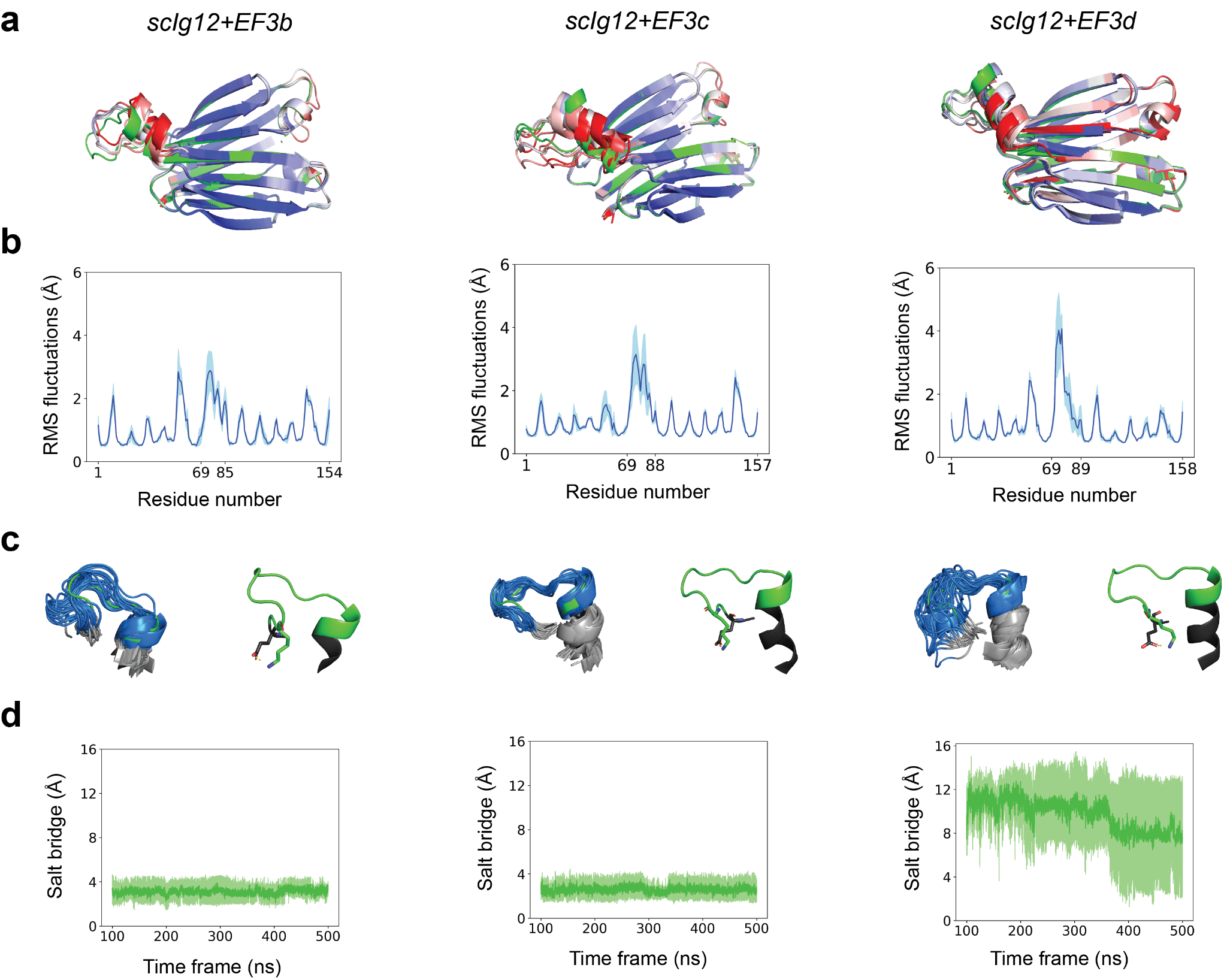


**Supplementary Figure 10.** **Computational characterization of functional loop scaffolding. a,** Remaining best performing functionalized designs (green) and their AlphaFold2 predictions colored by pLDDT (from red to blue increasing in pLDDT; scale 70 to 100). **b,** Averaged RMSFs. Y-axis indicates fluctuations in Angstroms for each residue. X-axis shows the first and last residues of the design and the grafted motif (central region). **c,** Representation of the grafted motifs (left). Ensemble of 30 conformations (10 per replicate) sampled during the molecular dynamics simulations every 50 ns (right). Black/Gray: Designed linkers. Green/Blue: EF-hand metal binding loop. Sticks are shown for the salt-bridging residues. **d,** Pairwise distances between the salt bridge forming residues in Angstroms (y-axis) as a matter of simulation time (x-axis). Source data are provided as a Source Data file.


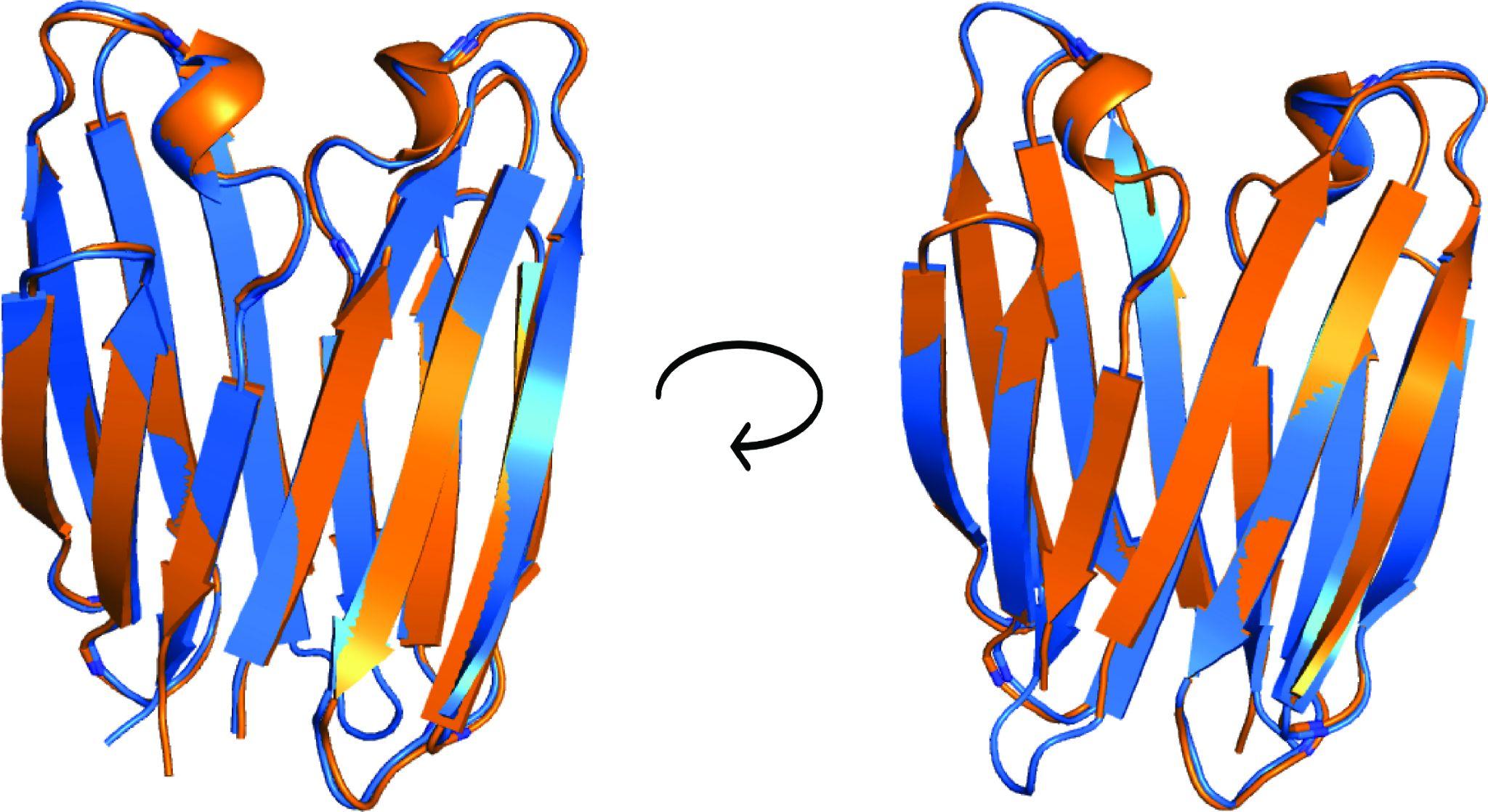


**Supplementary Figure 11. Comparison between the crystal structures of scIg12 (blue) and scIg12+EF3a (orange).** The structural agreement (Cα-RMSD of 0.7 Å) is very high despite the 15-residue functional insertion of scIg12+EF3a.

**
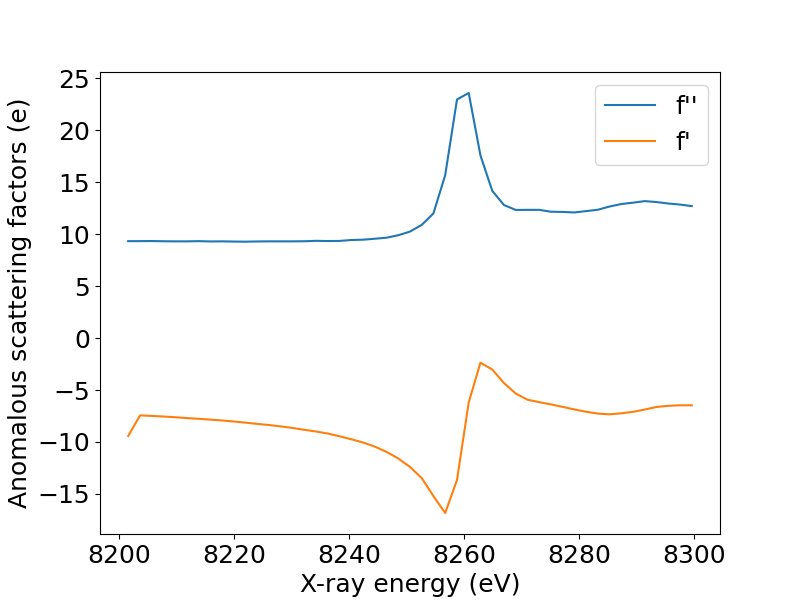
**

**Supplementary Figure 12. Anomalous X-ray scattering data for the crystal structure of scIg12+EF3a.** Experimental values of the anomalous scattering factors near the absorption edge of terbium (theoretical energy of 8252 eV).


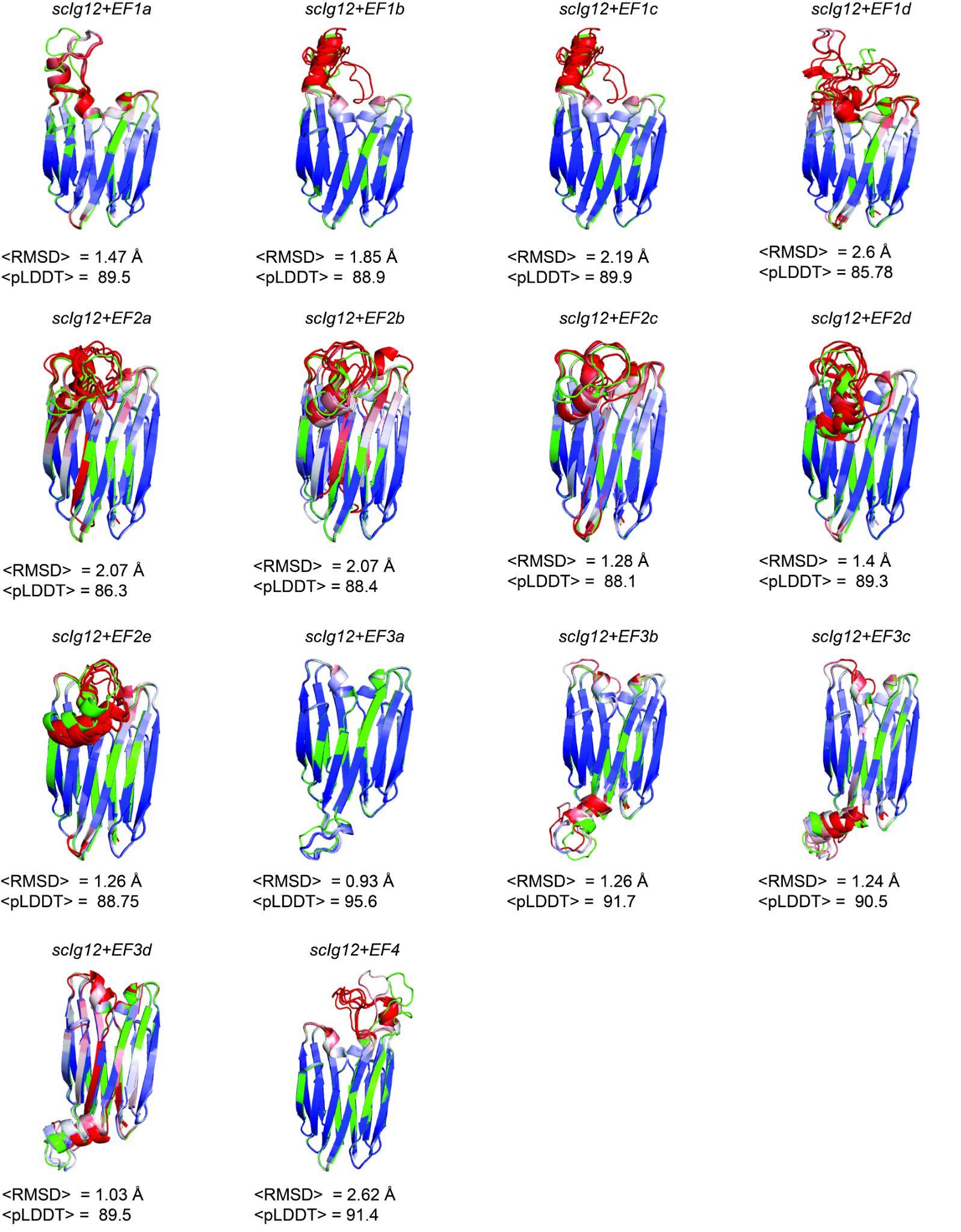


**Supplementary Figure 13. Computational designs and AlphaFold2 predictions of the selected mono-functionalized scIg12 scaffolds.** Superimposition of AF2 predictions colored by increasing pLDDT (from red to blue; scale 70 to 100) to their respective computational designs (green). For each design, average Cα-RMSD and pLDDT values are reported.


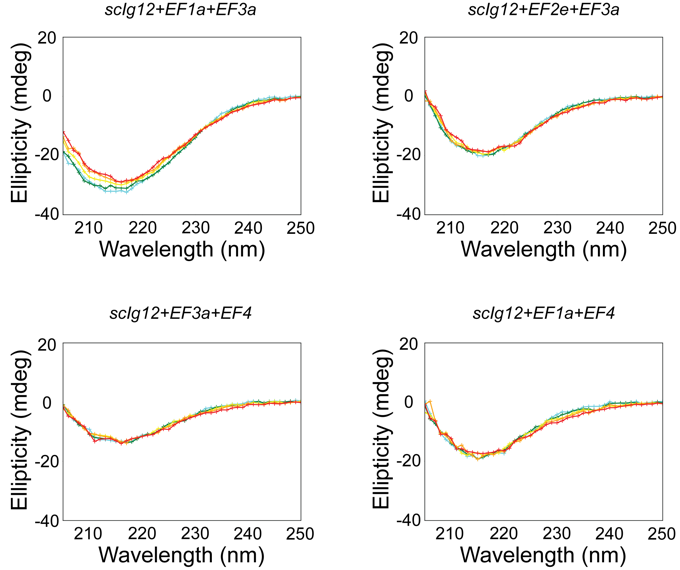


**Supplementary Figure 14. Far ultraviolet circular dichroism spectra at increasing temperature for the 4 designs with two functional loops.** Aqua: 25ºC, green: 40ºC, orange 60ºC and red: 90ºC. Source data are provided as a Source Data file.

**
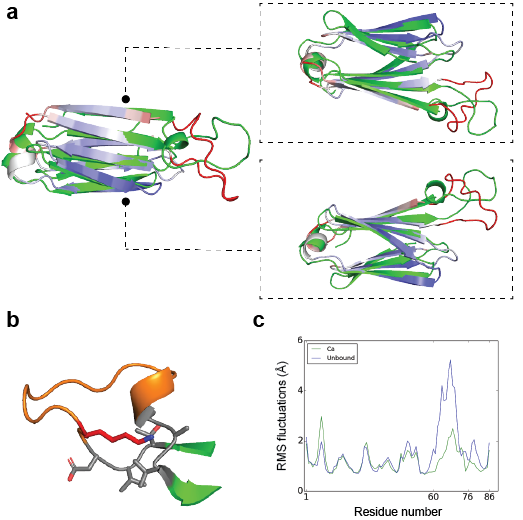
**

**Supplementary Figure 15. Computational design and characterization of a metal-binding loop on dIG8-CC**^8^**. a,** First AlphaFold2 model colored by increasing pLDDT (from red to blue; scale 70 to 100) superimposed to the design model (green). **b,** Closer view of the inserted fragment. In orange the EF-hand motif. In gray and sticks the designed linkers. The identified lysine (red) is not in contact with any other atom. **c,** RMSFs obtained from 300 ns molecular dynamics simulations in the presence and absence of calcium. The observed higher fluctuations in the absence of the ion suggest a low degree of loop preorganization. Source data are provided as a Source Data file.

**Supplementary Table 1.** Structural similarity metrics and confidence values of AlphaFold2 predictions for each of our selected parallel single-chain immunoglobulin dimers. RMSDs are calculated on Cα atoms.

1. Structural metrics for *scIg4a*

| **Prediction** | **RMSD (Å)** | | | | **pLDDT** |
| --- | --- | --- | --- | --- | --- |
|  | **Total** | **βα linker** | **α-helix** | **αβ linker** |  |
| *Rank 1* | 0.85 | 0.87 | 0.88 | 0.89 | 93.7 |
| *Rank 2* | 0.81 | 0.83 | 0.77 | 0.86 | 93.6 |
| *Rank 3* | 0.82 | 0.51 | 0.88 | 0.94 | 93.2 |
| *Rank 4* | 0.89 | 0.70 | 0.87 | 0.99 | 93.1 |
| *Rank 5* | 0.84 | 0.50 | 0.85 | 0.93 | 92.5 |

1. Structural metrics for *scIg14b*

| **Prediction** | **RMSD (Å)** | | | | **pLDDT** |
| --- | --- | --- | --- | --- | --- |
|  | **Total** | **βα linker** | **α-helix** | **αβ linker** |  |
| *Rank 1* | 0.86 | 0.59 | 0.87 | 0.81 | 93.2 |
| *Rank 2* | 1.10 | 0.77 | 1.07 | 0.90 | 92.1 |
| *Rank 3* | 1.09 | 0.81 | 0.99 | 0.93 | 91.7 |
| *Rank 4* | 0.96 | 0.71 | 0.97 | 0.95 | 91.6 |
| *Rank 5* | 1.01 | 0.58 | 0.93 | 0.99 | 90.1 |

1. Structural metrics for *scIg14c*

| **Prediction** | **RMSD (Å)** | | | | **pLDDT** |
| --- | --- | --- | --- | --- | --- |
|  | **Total** | **βα linker** | **α-helix** | **αβ linker** |  |
| *Rank 1* | 0.85 | 0.92 | 0.89 | 0.57 | 93.7 |
| *Rank 2* | 0.86 | 0.78 | 0.79 | 0.73 | 93.4 |
| *Rank 3* | 0.83 | 0.83 | 0.78 | 0.61 | 93.3 |
| *Rank 4* | 0.90 | 0.89 | 0.80 | 0.65 | 92.8 |
| *Rank 5* | 0.85 | 0.84 | 0.82 | 0.66 | 92.6 |

**Supplementary Table 2.** Size-exclusion chromatography combined with multi-angle scattering data. Samples were run on a Superdex 75 10/300 increase and were prepared in 30 mM Tris·HCl, 250 mM sodium chloride, pH 8 at 25 ºC.

| **Design name** | **Theoretical  Mw (kDa)** | **Estimated Mw (kDa)** |
| --- | --- | --- |
| scIg14b | 19.3 | 16.1 ± 0.2 |
| scIg12 | 18.4 | 20.6 ± 0.6 |
| scIg12+EF3a | 20.1 | 21.7 ± 0.9 |
| scIg12+EF1a+EF3a | 21.9 | 25.0 ± 0.3 |
| scIg12+EF2e+EF3a | 22.2 | 24.7 ± 0.1 |
| scIg12+EF3a+EF4 | 21.9 | 23.5 ± 1.2 |
| scIg12+EF1a+EF4 | 22.2 | 22.2 ± 0.1 |

**Supplementary Table 3.** Structural similarity metrics and confidence values of AlphaFold2 predictions for our antiparallel single-chain immunoglobulin dimer scIg12. RMSDs are calculated on Cα atoms.

| **Prediction** | **RMSD (Å)** | **pLDDT** |
| --- | --- | --- |
| *Rank 1* | 0.32 | 94.6 |
| *Rank 2* | 0.42 | 94.2 |
| *Rank 3* | 0.32 | 92.6 |
| *Rank 4* | 1.22 | 74.9 |
| *Rank 5* | 5.02 | 71.6 |

**Supplementary Table 4. Data collection and refinement statistics (molecular replacement)**

|  | scIg12 | scIg12+EF3a |
| --- | --- | --- |
| **Data collection** |  |  |
| Space group | P 43 21 2 | P 43 21 2 |
| Cell dimensions |  |  |
| *a*, *b*, *c* (Å) | 72.01, 72.01, 97.07 | 73.84, 73.84, 96.00 |
| α, β, γ (°) | 90.00 90.00 90.00 | 90.00 90.00 90.00 |
| Resolution (Å) | 48.53-2.8 (2.95-2.8)* | 96.25-2.8 (2.95-2.8)* |
| *R*_merge_ | 0.094 (3.698) | 0.106 (1.255) |
| *I* / σ*I* | 16.2 (0.7) | 15.4 (2.6) |
| Completeness (%) | 99.9 (99.9) | 100 (99.9) |
| Redundancy | 11.4 (12.1) | 16.2 (17.3) |
|  |  |  |
| **Refinement** |  |  |
| Resolution (Å) | 45.09 – 2.80 | 58.59 - 2.80 |
| No. reflections | 6712 | 6261 |
| *R*_work_ / *R*_free_ | 0.225 (0.284) | 0.202 (0.257) |
| No. atoms |  |  |
| Protein | 1137 | 1135 |
| Ligand/ion | 17 | 0 |
| Water | 6 | 9 |
| *B*-factors |  |  |
| Protein | 104 | 101 |
| Ligand/ion | 119 | 156 |
| Water | 99 | - |
| R.m.s. deviations |  |  |
| Bond lengths (Å) | 0.013 | 0.008 |
| Bond angles (°) | 1.25 | 1.675 |

*Values in parentheses are for highest-resolution shell.

**Supplementary Table 5.** Structural similarity metrics and confidence values of AlphaFold2 predictions for each of our selected mono-functionalized designs. RMSDs are calculated on Cα atoms.

1. Metrics for *scIg12+EF1a*

| **Prediction** | **RMSD (Å)** | | | **pLDDT** | | |
| --- | --- | --- | --- | --- | --- | --- |
|  | ***scIg12+EF1a*** | ***scIg12*** | ***EF1a*** | ***scIg12+EF1a*** | ***scIg12*** | ***EF1a*** |
| *Rank 1* | 1.53 | 0.29 | 1.23 | 91.4 | 93.2 | 78.9 |
| *Rank 2* | 1.41 | 0.25 | 1.28 | 91.2 | 92.7 | 80.6 |
| *Rank 3* | 1.57 | 0.33 | 1.24 | 90.3 | 92.4 | 74.9 |
| *Rank 4* | 1.43 | 0.34 | 1.24 | 88.0 | 90.1 | 73.2 |
| *Rank 5* | 1.43 | 0.30 | 1.25 | 86.8 | 88.9 | 71.8 |

1. Metrics for *scIg12+EF2e*

| **Prediction** | **RMSD (Å)** | | | **pLDDT** | | |
| --- | --- | --- | --- | --- | --- | --- |
|  | ***scIg12+EF2e*** | ***scIg12*** | ***EF2e*** | ***scIg12+EF2e*** | ***scIg12*** | ***EF2e*** |
| *Rank 1* | 1.04 | 0.18 | 1.22 | 89.6 | 94.8 | 57.3 |
| *Rank 2* | 1.10 | 0.21 | 1.27 | 89.2 | 94.2 | 58.5 |
| *Rank 3* | 1.23 | 0.27 | 0.80 | 89.1 | 93.6 | 61.8 |
| *Rank 4* | 1.37 | 0.32 | 0.86 | 88.8 | 92.4 | 67.3 |
| *Rank 5* | 1.54 | 0.4 | 0.99 | 86.8 | 89.8 | 68.8 |

1. Metrics for *scIg12+EF3a*

| **Prediction** | **RMSD (Å)** | | | **pLDDT** | | |
| --- | --- | --- | --- | --- | --- | --- |
|  | ***scIg12+EF3a*** | ***scIg12*** | ***EF3a*** | ***scIg12+EF3a*** | ***scIg12*** | ***EF3a*** |
| *Rank 1* | 0.97 | 0.18 | 1.17 | 96.3 | 96.5 | 93.7 |
| *Rank 2* | 0.97 | 0.21 | 1.16 | 95.9 | 96.1 | 93.5 |
| *Rank 3* | 0.91 | 0.16 | 1.14 | 95.8 | 96.1 | 92.5 |
| *Rank 4* | 0.99 | 0.17 | 1.14 | 95.1 | 95.5 | 91.4 |
| *Rank 5* | 0.81 | 0.18 | 1.15 | 95.0 | 95.5 | 90.2 |

1. Metrics for *scIg12+EF4*

| **Prediction** | **RMSD (Å)** | | | **pLDDT** | | |
| --- | --- | --- | --- | --- | --- | --- |
|  | ***scIg12+EF4*** | ***scIg12*** | ***EF4*** | ***scIg12+EF4*** | ***scIg12*** | ***EF4*** |
| *Rank 1* | 1.62 | 0.26 | 1.41 | 92.9 | 94.5 | 81.0 |
| *Rank 2* | 3.61 | 0.27 | 3.27 | 91.9 | 94.9 | 70.0 |
| *Rank 3* | 2.78 | 0.19 | 2.39 | 91.1 | 94.5 | 66.1 |
| *Rank 4* | 2.95 | 0.21 | 2.54 | 91.1 | 94.9 | 63.0 |
| *Rank 5* | 3.08 | 0.21 | 2.89 | 90.1 | 93.9 | 62.3 |

**Supplementary Table 6.** Structural similarity metrics and confidence values of AlphaFold2 predictions for each of our selected bi-functionalized designs. RMSDs are calculated on Cα atoms.

1. Structural metrics for *scIg12+EF1a+EF3a*

| **Prediction** | **RMSD (Å)** | | | **pLDDT** | | |
| --- | --- | --- | --- | --- | --- | --- |
|  | ***scIg12*** | ***EF1a*** | ***EF3a*** | ***scIg12*** | ***EF1a*** | ***EF3a*** |
| *Rank 1* | 0.45 | 1.79 | 1.18 | 90.2 | 65.2 | 84.9 |
| *Rank 2* | 0.38 | 1.25 | 1.19 | 84.5 | 64.5 | 83.8 |
| *Rank 3* | 2.40 | 1.43 | 1.15 | 81.9 | 69.0 | 77.5 |
| *Rank 4* | 1.59 | 2.48 | 1.50 | 79.5 | 58.5 | 71.9 |
| *Rank 5* | 1.14 | 2.39 | 0.99 | 75.5 | 64.0 | 58.8 |

1. Structural metrics for *scIg12+EF2e+EF3a*

| **Prediction** | **RMSD (Å)** | | | **pLDDT** | | |
| --- | --- | --- | --- | --- | --- | --- |
|  | ***scIg12*** | ***EF2e*** | ***EF3a*** | ***scIg12*** | ***EF2e*** | ***EF3a*** |
| *Rank 1* | 0.28 | 1.01 | 1.12 | 91.0 | 61.4 | 88.7 |
| *Rank 2* | 0.22 | 1.26 | 1.15 | 92.4 | 63.2 | 90.9 |
| *Rank 3* | 0.33 | 1.08 | 1.15 | 91.5 | 66.6 | 87.2 |
| *Rank 4* | 0.43 | 1.14 | 1.61 | 86.6 | 61.5 | 71.9 |
| *Rank 5* | 0.43 | 1.11 | 1.08 | 83.2 | 62.1 | 74.7 |

1. Structural metrics for *scIg12+EF3a+EF4*

| **Prediction** | **RMSD (Å)** | | | **pLDDT** | | |
| --- | --- | --- | --- | --- | --- | --- |
|  | ***scIg12*** | ***EF3a*** | ***EF4*** | ***scIg12*** | ***EF3a*** | ***EF4*** |
| *Rank 1* | 15.90 | 9.64 | 1.89 | 76.6 | 42.6 | 55.9 |
| *Rank 2* | 4.82 | 5.26 | 4.51 | 75.1 | 50.4 | 52.7 |
| *Rank 3* | 15.91 | 9.64 | 2.00 | 70.8 | 38.2 | 62.5 |
| *Rank 4* | 16.06 | 9.68 | 2.05 | 63.9 | 33.4 | 48.9 |
| *Rank 5* | 16.20 | 9.78 | 2.04 | 61.0 | 33.3 | 55.6 |

1. Structural metrics for *scIg12+EF1a+EF4*

| **Prediction** | **RMSD (Å)** | | | **pLDDT** | | |
| --- | --- | --- | --- | --- | --- | --- |
|  | ***scIg12*** | ***EF1a*** | ***EF4*** | ***scIg12*** | ***EF1a*** | ***EF4*** |
| *Rank 1* | 0.49 | 1.28 | 1.36 | 90.1 | 72.3 | 65.9 |
| *Rank 2* | 0.46 | 1.30 | 1.33 | 87.0 | 74.5 | 71.2 |
| *Rank 3* | 0.61 | 1.66 | 1.51 | 86.7 | 69.1 | 68.7 |
| *Rank 4* | 0.45 | 1.30 | 1.38 | 85.2 | 63.7 | 59.3 |
| *Rank 5* | 0.66 | 1.37 | 1.53 | 83.0 | 71.5 | 63.4 |

**Supplementary Table 7.** Structural similarity metrics and confidence values of AlphaFold2 predictions for dIG8-CC^8^ with a grafted EF-hand binding loop. RMSDs are calculated on Cα atoms.

| **Prediction** | **RMSD (Å)** | | | **pLDDT** | | |
| --- | --- | --- | --- | --- | --- | --- |
|  | **Total** | **Scaffold** | **Motif** | **Total** | **Scaffold** | **Motif** |
| *Rank 1* | 4.13 | 2.79 | 2.81 | 82.5 | 87.9 | 62.1 |
| *Rank 2* | 4.09 | 2.83 | 2.54 | 81.8 | 87.4 | 60.3 |
| *Rank 3* | 3.78 | 2.69 | 2.26 | 81.6 | 87.8 | 58.4 |
| *Rank 4* | 4.14 | 2.85 | 3.12 | 80.4 | 86.5 | 57.5 |
| *Rank 5* | 4.14 | 2.62 | 3.62 | 80.3 | 86.3 | 57.5 |

**Supplementary Table 8.** Designed sequences of the antiparallel (scIg12) and parallel (scIg14) single-chain immunoglobulin dimers. Shaded sequences correspond to the experimentally tested proteins.

| **Design** | **Sequence** |
| --- | --- |
| scIg12 | GRVEVRVEFEGDKMRVRLRNDSSTPVEVHIKVGDEKRTVTVNPGEEVEVTFSANDPHKFNRPQFTIEWGGGGRVEVRVEFEGDKMRVRLRNDSSTPVEVHIKVGDEKRTVTVNPGEEVEVTFSANDPHKFNRPQFTIEWG |
| scIg14a | TETIEVRVDNGRVRVRNGTDRPIRVRVTAGGETREYTVNPGTELEVELSPEQQNQAIVVIHIGNEVFMFVLARDEEWVKRAEKLAEELNVRILVIVLNGRVRVRNGTDRPMRVRVTAGGETREYTVNPGTELEVELSPEQQNNAEVEVEVGNRKWRFQLG |
| scIg14b | RIEVRVDNGRVRVRNGTDRPIRVRVTAGGETREYTVNPGTELEVELSPEQQNQAIVVVHIGNRVFMWVLARDEEWVKRAEKLAEELNVRILVIVLNGRVRVRNGTDRPMRVRVTAGGETREYTVNPGTELEVELSPEQQNNAEVEVEVGNEKWRFQLG |
| scIg14c | TQTIEVRVDNGRVRVRNGTDRPIRVRVTAGGETREYTVNPGTELEVELSPEQQNNAMVEVQVGNEIVFFILAHNEELAKRWWEEAKQRAKILVMVLNGRVRVRNGTDRPMRVRVTAGGETREYTVNPGTELEVELSPEQQNNAEVEVEVGNNKYRFQLG |
| scIg14d | RIEVRVDNGRVRVRNGTDRPCRVRVTAGGETREYTVNPGTELEVELSPEQQNNALVVVTCGNETYVFILAHDPEEAKKWHELAERYNPKIIVIVVNGRVRVRNGTDRPCRVRVTAGGETREYTVNPGTELEVELSPEQQNNAEVEVECGNEKYRFQLG |
| scIg14e | RIEVRVDNGRVRVRNGTDRPCRVRVTAGGETREYTVNPGTELEVELSPEQQNNAVVEVQCGNEIYIFILATDPRLAKEWWEKAKQKAKILVIVVNGRVRVRNGTDRPCRVRVTAGGETREYTVNPGTELEVELSPEQQNNAEVEVECGNEKYRFQLG |
| scIg14f | RIEVRVDNGRVRVRNGTDRPCRVRVTAGGETREYTVNPGTELEVELSPEQQNNALVEVECGNERYVFILATDEETAKEWIKKAHEIADIVVVVLNGRVRVRNGTDRPCRVRVTAGGETREYTVNPGTELEVELSPEQQNNAEVEVECGNEKYRFQLG |
| scIg14g | RIEVRVDNGRVRVRNGTDRPCRVRVTAGGETREYTVNPGTELEVELSPEQQNNAMVEVHCGNEYYFFILAHNPELAEKWWQKAQKHAKIVVMVLNGRVRVRNGTDRPCRVRVTAGGETREYTVNPGTELEVELSPEQQNNAEVEVECGNEKYRFQLG |
| scIg14h | RIEVRVDNGRVRVRNGTDRPCRVRVTAGGETREYTVNPGTELEVELSPEQQNNAIVTVHCGNEYYLFVLAHDERIAEKAIEHAERLGARIIVVVINGRVRVRNGTDRPCRVRVTAGGETREYTVNPGTELEVELSPEQQNNAEVEVECGNEKYRFQLG |
| scIg14i | RIEVRVDNGRVRVRNGTDRPCRVRVTAGGETREYTVNPGTELEVELSPEQQNNAIVTVHCGNEVYMFVLATDPEFAKLAEKWAEEMNVKIIVIVLNGRVRVRNGTDRPCRVRVTAGGETREYTVNPGTELEVELSPEQQNNAEVEVECGNEKYRFQLG |
| scIg14j | RIEVRVDNGRVRVRNGTDRPCRVRVTAGGETREYTVNPGTELEVELSPEQQNNALVIVTCGNETYVFMLARDEKAMKEFQKEAEKYNPKIIVMVLNGRVRVRNGTDRPCRVRVTAGGETREYTVNPGTELEVELSPEQQNNAEVEVECGNEKYRFQLG |
| scIg14k | RIEVRVDNGRVRVRNGTDRPCRVRVTAGGETREYTVNPGTELEVELSPEQQNNAIVVVHCGNEIYVFILVTSEEARKHFEKIARELKPKILVMVHNGRVRVRNGTDRPCRVRVTAGGETREYTVNPGTELEVELSPEQQNNAEVEVECGNEKYRFQLG |
| scIg14l | RIEVRVDNGRVRVRNGTDRPCRVRVTAGGETREYTVNPGTELEVELSPEQQNNAVVVVHCGNEYYVFILATDEEQRKEFEKVAEKLNAKILVVVVNGRVRVRNGTDRPCRVRVTAGGETREYTVNPGTELEVELSPEQQNNAEVEVECGNEKYRFQLG |
| scIg14m | RIEVRVDNGRVRVRNGTDRPCRVRVTAGGETREYTVNPGTELEVELSPEQQNNALVVVTCGNETYVFILAHDPEEAKKWHELAERYNPKIIVIVVNGRVRVRNGTDRPCRVRVTAGGETREYTVNPGTELEVELSPEQQNNAEVEVECGNEKYRFQLG |
| scIg14n | RIEVRVDNGRVRVRNGTDRPCRVRVTAGGETREYTVNPGTELEVELSPEQQNNAIVVVHCGNEWYMFVLARDEEWVKRAEKLAEELNVRILVIVLNGRVRVRNGTDRPCRVRVTAGGETREYTVNPGTELEVELSPEQQNNAEVEVECGNEKYRFQLG |

**Supplementary Table 9.** Designed sequences of the face-to-face single-chain immunoglobulin dimers.

| **Design** | **Sequence** |
| --- | --- |
| scIgFFa | RIEVRVDNGRVRVRNGTDRPCTVHFVWGGEIRTYTVNPGTELEVELSPEHQNNMAVVIVCGDEVYVWATEHPGIYIVLHDGILVIIITTDRPCRVRVTAGGETREYTLNPHTYMVLHLSPEQQNNAEVEVECGNEKVRYL |
| scIgFFb | RIEVRVDNGRVRVRNGTDRPCVVHFIWGGEIREYTVNPGTELEVELSPEHQNNMVVVIVCGDEVYVWFTEHPQIAIVLHDGILVIVITTDRPCRVRVTAGGETREYTLNPHTYMVLHLSPEQQNNAEVEVECGNEKKRYL |
| scIgFFc | RIEVRVDNGRVRVRNGTDRPCTVYVIFGGETRVYQVNPGTELEVELSPEHQNDVVIVIFCGSEVYVWATPNPLLAVVLHNGTLVIVLTTDRPCRVRVTAGGETREYTVNPNTVMVLQLSPEQQNNAEVEVECGNEKYRYL |
| scIgFFd | RIEVRVDNGRVRVRNGTDRPCTVYVIFGGELREYQVNPGTELEVELSPEHQNRVVIIIFCGSEVYVWATPNPLLAVVLHNGTLVIVLTTDRPCRVRVTAGGETREYTVNPNTVMVLQLSPEQQNNAEVEVECGNEKFRYL |
| scIgFFe | RIEVRVDNGRVRVRNGTDRPCTVIFYWGGEQRVYTVNPGTELEVELSPEYQNRMVVVIICGDEVYVWTTDQPMIAVILHDGVLVIVLTTDRPCRVRVTAGGETREYTLNPRTVMVVRLSPEQQNNAEVEVECGNEKYRFL |
| scIgFFf | RIEVRVDNGRVRVRNGTDRPCTVIFYWGGEQRVYTVNPGTELEVELSPEYQNKMVVVIICGDEVYVWTTDQPMIAVVLHDGVLVIVLTTDRPCRVRVTAGGETREYTLNPKTVMVVRLSPEQQNNAEVEVECGNEKYRFL |
| scIgFFg | RIEVRVDNGRVRVRNGTDRPCTVIFYWGGEQRVYTVNPGTELEVELSPEYQNKMVVVIICGDEVYVWTTDQPMIAVILHDGVLVIVLTTDRPCRVRVTAGGETREYTLNPKTVMVVRLSPEQQNNAEVEVECGNEKYRFL |
| scIgFFh | RIEVRVDNGRVRVRNGTDRPCTVVVVAGGEHRTYQVNPGTELEVELSPETQNDVVVVIFCSEEVYVFSTDHPRIVVVLHDGTLVIVINTDRPCRVRVTAGGETREYTVNPNTYMVVRLSPEQQNNAEVEVECGNEKWRYL |
| scIgFFi | RIEVRVDNGRVRVRNGTDRPCTVIFVSGGETRVYQVNPGTELEVELSPETQNDVVIVIICGSEVHVWSTPTKRLVIVLHDGTLVIFINTDRPCRVRVTAGGETREYTLNPNTYMVLQLSPEQQNNAEVEVECGNEKYRYL |
| scIgFFj | RIEVRVDNGRVRVRNGTDRPCTVIFVSGGEVRTYQVNPGTELEVELSPETQNDVVIVIICGSEVHVWSTPTKRLVIVLHEGTLVIFINTDRPCRVRVTAGGETREYTLNPNTYMVLQLSPEQQNNAEVEVECGNEKYRYL |
| scIgFFk | RIEVRVDNGRVRVRNGTDRPCTVHVVSGGEVRTYTVNPGTELEVELSPEHQNRVVVVIICSDEVYVWSTDDPEIVVVLHDGILVIVITTDRPCRVRVTAGGETREYTVNPGTYWVVRLSPEQQNNAEVEVECGNEKYRYL |
| scIgFFl | RIEVRVDNGRVRVRNGTDRPCTVTVISGGEVRTYTVNPGTELEVELSPEHQNRVVVVIICSDEVHVWMTDDPLIAVVLHDGTLVIVITTDRPCRVRVTAGGETREYTVNPGTYWVVKLSPEQQNNAEVEVECGNEKYRYL |
| scIgFFm | RIEVRVDNGRVRVRNGTDRPCVVIVASGGEIREYTVNPGTELEVELSPEHQNKVVVIIICSDEVHVWMTDDPEIVVVLHEGTLVIVITTDRPCRVRVTAGGETREYTVNPGTYWVVKLSPEQQNNAEVEVECGNEKYRYL |
| scIgFFn | RIEVRVDNGRVRVRNGTDRPCIVIFIWGHEIRHYTVNPGTELEVELSPEHQNQMVVVIICGSEVYVWMTEHPMIVIVLVNGILIIVIHTDRPCRVRVTAGGETREYTLNPKTYMVLHLSPEQQNNAEVEVECGNEKVRYL |
| scIgFFo | RIEVRVDNGRVRVRNGTDRPCTVFVVFGNEYRQYQVNPGTELEVELSPEQQNHVIIIIFCGSEVYVWFMPNPLLVIVLYQGILVIVLTTDRPCRVRVTAGGETREYTVNPNTVMILQLSPEQQNNAEVEVECGNEKYRYL |
| scIgFFp | RIEVRVDNGRVRVRNGTDRPCIVIFSWGNEQRTYTVNPGTELEVELSPEYQNHMVVVIICGEEVYVWITDQPMIVVILYNGILIIILSTDRPCRVRVTAGGETREYTLNPKTVMIVKLSPEQQNNAEVEVECGNEKYRFL |
| scIgFFq | RIEVRVDNGRVRVRNGTDRPCIVVVVAGNEYRQYQVNPGTELEVELSPETQNRVVVVIFCSEEVYVFWTDHPMIVIILINGILVIIILTDRPCRVRVTAGGETREYTVNPNTYMTVTLSPEQQNNAEVEVECGNEKWRYL |
| scIgFFr | RIEVRVDNGRVRVRNGTDRPCIVIFVWGGEERRYQVNPGTELEVELSPETQNKVVIIIICGSEVYVWWTPTKMLVIVLVNGILVILILTDRPCRVRVTAGGETREYTLNPQTYMVLQLSPEQQNNAEVEVECGNEKYRYL |
| scIgFFs | RIEVRVDNGRVRVRNGTDRPCIVIVVFGGEYRTYTVNPGTELEVELSPEYQNKVVVIIICSEEVYVWMSDDDLIVIYLYNGILIIVILTDRPCRVRVTAGGETREYTVNPHTYWVVKLSPEQQNNAEVEVECGNEKYRYL |

**Supplementary Table 10.** Sequences of the mono- and bi-functionalized single-chain immunoglobulin dimers. Shaded sequences correspond to the experimentally tested proteins.

| **Design** | **Sequence** |
| --- | --- |
| scIg12+EF1a | GRVEVRVEFEPIEDKDGDGYISAAEAAAAKMRVRLRNDSSTPVEVHIKVGDEKRTVTVNPGEEVEVTFSANDPHKFNRPQFTIEWGGGGRVEVRVEFEGDKMRVRLRNDSSTPVEVHIKVGDEKRTVTVNPGEEVEVTFSANDPHKFNRPQFTIEWG |
| scIg12+EF1b | GRVEVRVEFEEGKDKDGDGYISAAEKEEAKKDAKMRVRLRNDSSTPVEVHIKVGDEKRTVTVNPGEEVEVTFSANDPHKFNRPQFTIEWGGGGRVEVRVEFEGDKMRVRLRNDSSTPVEVHIKVGDEKRTVTVNPGEEVEVTFSANDPHKFNRPQFTIEWG |
| scIg12+EF1c | GRVEVRVEFETPQDKDGDGYISAAELEEAAKDAKMRVRLRNDSSTPVEVHIKVGDEKRTVTVNPGEEVEVTFSANDPHKFNRPQFTIEWGGGGRVEVRVEFEGDKMRVRLRNDSSTPVEVHIKVGDEKRTVTVNPGEEVEVTFSANDPHKFNRPQFTIEWG |
| scIg12+EF1d | GRVEVRVEFESDSLEDKDGDGYISAAEAAGKMRVRLRNDSSTPVEVHIKVGDEKRTVTVNPGEEVEVTFSANDPHKFNRPQFTIEWGGGGRVEVRVEFEGDKMRVRLRNDSSTPVEVHIKVGDEKRTVTVNPGEEVEVTFSANDPHKFNRPQFTIEWG |
| scIg12+EF2a | GRVEVRVEFEGDKMRVRLRNDSSTPVEVHIKVLGDKDGDGYISAAEAKQSEKRTVTVNPGEEVEVTFSANDPHKFNRPQFTIEWGGGGRVEVRVEFEGDKMRVRLRNDSSTPVEVHIKVGDEKRTVTVNPGEEVEVTFSANDPHKFNRPQFTIEWG |
| scIg12+EF2b | GRVEVRVEFEGDKMRVRLRNDSSTPVEVHIKVSGDKDGDGYISAAEDKQGEKRTVTVNPGEEVEVTFSANDPHKFNRPQFTIEWGGGGRVEVRVEFEGDKMRVRLRNDSSTPVEVHIKVGDEKRTVTVNPGEEVEVTFSANDPHKFNRPQFTIEWG |
| scIg12+EF2c | GRVEVRVEFEGDKMRVRLRNDSSTPVEVHIKVSGDKDGDGYISAAEAADAEKRTVTVNPGEEVEVTFSANDPHKFNRPQFTIEWGGGGRVEVRVEFEGDKMRVRLRNDSSTPVEVHIKVGDEKRTVTVNPGEEVEVTFSANDPHKFNRPQFTIEWG |
| scIg12+EF2d | GRVEVRVEFEGDKMRVRLRNDSSTPVEVHIKVGADKDGDGYISAAEAEDGEKRTVTVNPGEEVEVTFSANDPHKFNRPQFTIEWGGGGRVEVRVEFEGDKMRVRLRNDSSTPVEVHIKVGDEKRTVTVNPGEEVEVTFSANDPHKFNRPQFTIEWG |
| scIg12+EF2e | GRVEVRVEFEGDKMRVRLRNDSSTPVEVHIKVGGDKDGDGYISAAEAKDAEKDAEKRTVTVNPGEEVEVTFSANDPHKFNRPQFTIEWGGGGRVEVRVEFEGDKMRVRLRNDSSTPVEVHIKVGDEKRTVTVNPGEEVEVTFSANDPHKFNRPQFTIEWG |
| scIg12+EF3a | GRVEVRVEFEGDKMRVRLRNDSSTPVEVHIKVGDEKRTVTVNPGEEVEVTFSANDPHKFNRPQFTIEWKDDKDGDGYISAAEKGRVEVRVEFEGDKMRVRLRNDSSTPVEVHIKVGDEKRTVTVNPGEEVEVTFSANDPHKFNRPQFTIEWG |
| scIg12+EF3b | GRVEVRVEFEGDKMRVRLRNDSSTPVEVHIKVGDEKRTVTVNPGEEVEVTFSANDPHKFNRPQFTIEWLEDKDGDGYISAAEKEQGRVEVRVEFEGDKMRVRLRNDSSTPVEVHIKVGDEKRTVTVNPGEEVEVTFSANDPHKFNRPQFTIEWG |
| scIg12+EF3c | GRVEVRVEFEGDKMRVRLRNDSSTPVEVHIKVGDEKRTVTVNPGEEVEVTFSANDPHKFNRPQFTIEWLEDKDGDGYISAAEKEAAKNGRVEVRVEFEGDKMRVRLRNDSSTPVEVHIKVGDEKRTVTVNPGEEVEVTFSANDPHKFNRPQFTIEWG |
| scIg12+EF3d | GRVEVRVEFEGDKMRVRLRNDSSTPVEVHIKVGDEKRTVTVNPGEEVEVTFSANDPHKFNRPQFTIEWLEDKDGDGYISAAEKEQAAKEGRVEVRVEFEGDKMRVRLRNDSSTPVEVHIKVGDEKRTVTVNPGEEVEVTFSANDPHKFNRPQFTIEWG |
| scIg12+EF4 | GRVEVRVEFEGDKMRVRLRNDSSTPVEVHIKVGDEKRTVTVNPGEEVEVTFSANDPHKFNRPQFTIEWGGGGRVEVRVEFEPIEDKDGDGYISAAEAAAAKMRVRLRNDSSTPVEVHIKVGDEKRTVTVNPGEEVEVTFSANDPHKFNRPQFTIEWG |
| scIg12+EF1a+EF3a | GRVEVRVEFEPIEDKDGDGYISAAEAAAAKMRVRLRNDSSTPVEVHIKVGDEKRTVTVNPGEEVEVTFSANDPHKFNRPQFTIEWKDDKDGDGYISAAEKGRVEVRVEFEGDKMRVRLRNDSSTPVEVHIKVGDEKRTVTVNPGEEVEVTFSANDPHKFNRPQFTIEWG |
| scIg12+EF2e+EF3a | GRVEVRVEFEGDKMRVRLRNDSSTPVEVHIKVGGDKDGDGYISAAEAKDAEKDAEKRTVTVNPGEEVEVTFSANDPHKFNRPQFTIEWKDDKDGDGYISAAEKGRVEVRVEFEGDKMRVRLRNDSSTPVEVHIKVGDEKRTVTVNPGEEVEVTFSANDPHKFNRPQFTIEWG |
| scIg12+EF1a+EF4 | GRVEVRVEFEPIEDKDGDGYISAAEAAAAKMRVRLRNDSSTPVEVHIKVGDEKRTVTVNPGEEVEVTFSANDPHKFNRPQFTIEWGGGGRVEVRVEFEPIEDKDGDGYISAAEAAAAKMRVRLRNDSSTPVEVHIKVGDEKRTVTVNPGEEVEVTFSANDPHKFNRPQFTIEWG |
| scIg12+EF3a+EF4 | GRVEVRVEFEGDKMRVRLRNDSSTPVEVHIKVGDEKRTVTVNPGEEVEVTFSANDPHKFNRPQFTIEWKDDKDGDGYISAAEKGRVEVRVEFEPIEDKDGDGYISAAEAAAAKMRVRLRNDSSTPVEVHIKVGDEKRTVTVNPGEEVEVTFSANDPHKFNRPQFTIEWG |

**Supplementary references**

1. Jumper, J. *et al.* Highly accurate protein structure prediction with AlphaFold. *Nature* **596**, 583–589 (2021).

2. Lin, Y.-R. *et al.* Control over overall shape and size in de novo designed proteins. *Proc. Natl. Acad. Sci.* **112**, E5478–E5485 (2015).

3. Leman, J. K. *et al.* Macromolecular modeling and design in Rosetta: recent methods and frameworks. *Nat. Methods* **17**, 665–680 (2020).

4. Bradley, P., Misura, K. M. S. & Baker, D. Toward High-Resolution de Novo Structure Prediction for Small Proteins. *Science* **309**, 1868–1871 (2005).

5. Varadi, M. *et al.* AlphaFold Protein Structure Database: massively expanding the structural coverage of protein-sequence space with high-accuracy models. *Nucleic Acids Res.* **50**, D439–D444 (2022).

6. van Kempen, M. *et al.* Fast and accurate protein structure search with Foldseek. *Nat. Biotechnol.* (2023) doi:10.1038/s41587-023-01773-0.

7. Zhang, Y. & Skolnick, J. TM-align: a protein structure alignment algorithm based on the TM-score. *Nucleic Acids Res.* **33**, 2302–2309 (2005).

8. Chidyausiku, T. M. *et al.* De novo design of immunoglobulin-like domains. *Nat. Commun.* **13**, 5661 (2022).
