# Supplementary material for: Single-chain dimers from de novo immunoglobulins as robust scaffolds for multiple binding loops
Source: Nat Commun. 2023 Sep 23;14:5939. doi: 10.1038/s41467-023-41717-5 (PMC10517939; doi:10.1038/s41467-023-41717-5)
Supplement: Supplementary file 2 — Reporting Summary [file 41467_2023_41717_MOESM2_ESM.pdf]

nature portfolio

Corresponding author(s): Enrique Marcos

Last updated by author(s): Sep 14, 2023

Reporting Summary

Nature Portfolio wishes to improve the reproducibility of the work that we publish. This form provides structure for consistency and transparency in reporting. For further information on Nature Portfolio policies, see our Editorial Policies and the Editorial Policy Checklist.

Statistics

For all statistical analyses, confirm that the following items are present in the figure legend, table legend, main text, or Methods section.

n/a	Confirmed	
☐	☒	The exact sample size (*n*) for each experimental group/condition, given as a discrete number and unit of measurement
☐	☒	A statement on whether measurements were taken from distinct samples or whether the same sample was measured repeatedly
☒	☐	The statistical test(s) used AND whether they are one- or two-sided<br>*Only common tests should be described solely by name; describe more complex techniques in the Methods section.*
☒	☐	A description of all covariates tested
☒	☐	A description of any assumptions or corrections, such as tests of normality and adjustment for multiple comparisons
☐	☒	A full description of the statistical parameters including central tendency (e.g. means) or other basic estimates (e.g. regression coefficient) AND variation (e.g. standard deviation) or associated estimates of uncertainty (e.g. confidence intervals)
☒	☐	For null hypothesis testing, the test statistic (e.g. *F*, *t*, *r*) with confidence intervals, effect sizes, degrees of freedom and *P* value noted<br>*Give P values as exact values whenever suitable.*
☒	☐	For Bayesian analysis, information on the choice of priors and Markov chain Monte Carlo settings
☒	☐	For hierarchical and complex designs, identification of the appropriate level for tests and full reporting of outcomes
☒	☐	Estimates of effect sizes (e.g. Cohen's *d*, Pearson's *r*), indicating how they were calculated

Our web collection on statistics for biologists contains articles on many of the points above.

Software and code

Policy information about availability of computer code

Data collection	The Rosetta molecular modeling software and its python interface PyRosetta were used to perform protein design calculations. Rosetta and PyRosetta are freely available for academic users. The Rosetta build used was: 2020.36.post.dev+22.master.842d147e4b9. The Pyrosetta build used was: v2020.37-dev61417-0-g3ba1aaa.<br>Structure prediction was also done with AlphaFold2 using the locally installed interface provided by LocalColabFold.<br>Sequence design calculations were also performed with ProteinMPNN (https://github.com/dauparas/ProteinMPNN.git)<br>Deep-network hallucination for structure diversification was performed with the oligomer hallucination code (https://github.com/bwicky/oligomer_hallucination)<br>Search for structural analogs of the designs was done using the Foldseek server (https://search.foldseek.com)
Data analysis	Protein structure analysis was done with custom python scripts, which have been provided with the Supplementary Datasets. These scripts use PyRosetta (v2020.37-dev61417-0-g3ba1aaa), which is freely available for academic users.<br>Sequence clustering was performed with mmseqs2 (version 13.45111)<br>Network representation of protein structure clustering was performed with Cytoscape (v3.10.0)<br>X-ray crystallography data were analyzed as described in the methods section with: Xds (Version Feb. 5, 2021), Refmac (v5.8.0258), Pointless (v1.12.8), Molrep (v11.7.02), Buster (v2.10.4) and Coot (v0.9.5)<br>Data analyses and graphical representations were done with python (v3.8), numpy (v1.19.1) and matplotlib (v3.3.1)<br>Visualization and image rendering of protein structures was done with Pymol (v2.4.0)<br>Analysis of size-exclusion chromatography with multi-angle light scattering data was done with ASTRA v7.2

For manuscripts utilizing custom algorithms or software that are central to the research but not yet described in published literature, software must be made available to editors and reviewers. We strongly encourage code deposition in a community repository (e.g. GitHub). See the Nature Portfolio guidelines for submitting code & software for further information.

Data

Policy information about availability of data

All manuscripts must include a data availability statement. This statement should provide the following information, where applicable:

- Accession codes, unique identifiers, or web links for publicly available datasets
- A description of any restrictions on data availability
- For clinical datasets or third party data, please ensure that the statement adheres to our policy

Coordinates and structure factors have been deposited in the Research Collaboratory for Structural Bioinformatics Protein Data Bank with the accession codes 8BL3 [https://www.rcsb.org/structure/8bl3] (design sclg12), [https://www.rcsb.org/structure/8bl6] 8BL6 (design sclg12+EF3a). The designed protein structures with high-confident AlphaFold2 predictions, including those experimentally tested, are available as Supplementary Dataset 1 along with their AlphaFold2 predictions (5 models), and their corresponding sequences are provided in Supplementary Table 8, 9 and 10. X-ray crystallography statistics are provided as Supplementary Table 4. The AlphaFold Protein Structure database used for structural analysis is freely available (https://alphafold.ebi.ac.uk). Previously published structures from the Protein Data Bank that were referenced throughout the manuscript are freely available: 5YD3 [https://www.rcsb.org/structure/5yd3], 7SKO [https://www.rcsb.org/structure/7sko], 7SKP [https://www.rcsb.org/structure/7skp], 4JOX [https://www.rcsb.org/structure/4jox], 1NKF [https://www.rcsb.org/structure/1nkf]. Other data are available from the corresponding author upon request. Source data are provided with this paper.

Field-specific reporting

Please select the one below that is the best fit for your research. If you are not sure, read the appropriate sections before making your selection.

☒ Life sciences ☐ Behavioural & social sciences ☐ Ecological, evolutionary & environmental sciences

For a reference copy of the document with all sections, see nature.com/documents/nr-reporting-summary-flat.pdf

Life sciences study design

All studies must disclose on these points even when the disclosure is negative.

Sample size	We have computationally designed hundreds of proteins and obtained 49 designs with highly confident predicted structures. Among them, we have experimentally tested 9 designed proteins that were representative for different structural features and functional motifs described in the paper. All experimentally tested proteins worked successfully. This number was considered sufficient to explore the different design families considered in this study.
Data exclusions	No data was excluded
Replication	Circular dichroism and SEC-MALS were done once or twice (all replicates were successful). The binding luminescence experiments were successfully repeated three or four times at multiple concentrations to improve statistic value.
Randomization	Randomization was not relevant to this work. All tested proteins received identical treatment.
Blinding	Blinding was not relevant to this work, since all tested proteins followed the same experimental procedures.

Reporting for specific materials, systems and methods

We require information from authors about some types of materials, experimental systems and methods used in many studies. Here, indicate whether each material, system or method listed is relevant to your study. If you are not sure if a list item applies to your research, read the appropriate section before selecting a response.

Materials & experimental systems

n/a	Involved in the study	
☒	☐	Antibodies
☒	☐	Eukaryotic cell lines
☒	☐	Palaeontology and archaeology
☒	☐	Animals and other organisms
☒	☐	Human research participants
☒	☐	Clinical data
☒	☐	Dual use research of concern

Methods

n/a	Involved in the study	
☒	☐	ChIP-seq
☒	☐	Flow cytometry
☒	☐	MRI-based neuroimaging
