# Supplementary figures and images for: Single-chain dimers from de novo immunoglobulins as robust scaffolds for multiple binding loops
Source: Nat Commun. 2023 Sep 23;14:5939. doi: 10.1038/s41467-023-41717-5 (PMC10517939; doi:10.1038/s41467-023-41717-5)

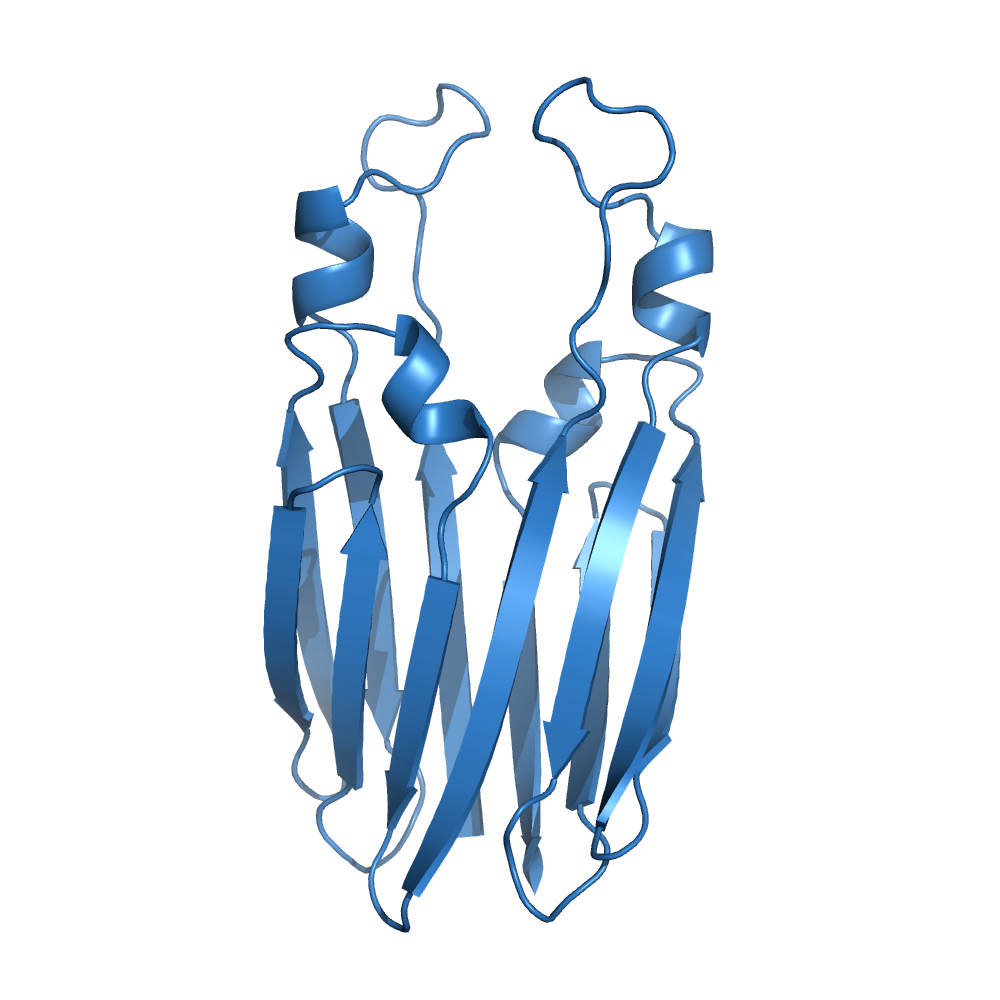

Supplement: Supplementary file 3 — Supplementary Data 1 [file 41467_2023_41717_MOESM3_ESM.zip › design_models/scIg+EF/scIg12+EF1a+EF4/scIg.png]
