# Supplementary material for: Single-chain dimers from de novo immunoglobulins as robust scaffolds for multiple binding loops
Source: Nat Commun. 2023 Sep 23;14:5939. doi: 10.1038/s41467-023-41717-5 (PMC10517939; doi:10.1038/s41467-023-41717-5)
Supplement: Supplementary file 4 — Description of Additional Supplementary Files [file 41467_2023_41717_MOESM4_ESM.docx]

Description of additional supplementary files

**File name:** Supplementary Data 1

**Description:** Designed protein structures with high-confident AlphaFold2 predictions and custom python scripts for design and analysis.
